# Group and partnered dance for people living with dementia: an overview of intervention design and measurement considerations

**DOI:** 10.3389/fpsyg.2025.1500688

**Published:** 2025-01-30

**Authors:** Deborah A. Jehu, Judith Bek, Crystal Bennett, Madeleine E. Hackney

**Affiliations:** ^1^Department of Community & Behavioral Health Sciences, Institute of Public and Preventative Health, Augusta University, Augusta, GA, United States; ^2^School of Psychology, University College Dublin, Dublin, Ireland; ^3^Centre for Motor Control, Faculty of Kinesiology & Physical Education, University of Toronto, Toronto, ON, Canada; ^4^College of Health Professions, University of Montevallo, Montevallo, AL, United States; ^5^Emory University School of Medicine Department of Medicine, Division of Geriatrics and Gerontology & Department of Rehabilitation, Atlanta, GA, United States; ^6^Atlanta VA Center for Visual and Neurocognitive Rehabilitation, Decatur, GA, United States; ^7^Birmingham/Atlanta VA Geriatric Research Education and Clinical Center, Decatur, GA, United States

**Keywords:** dementia, dance, intervention, exercise, cognitive decline, mild cognitive impairment, physical activity

## Introduction

Dementia is a growing global public health priority, with 55 million people living with dementia (PLWD) worldwide (WHO, 2023). Approximately 45%−60% of dementia cases are preventable through modifiable risk factors (Calandri et al., 2024; Livingston et al., 2024). Much of the research on dementia focuses on prevention through lifestyle interventions, such as exercise (Kivipelto et al., 2020). Considerably less research has addressed treating dementia symptoms through lifestyle interventions, as PLWD are often excluded from intervention studies due to ineligibility criteria. Interventions must be tailored for PLWD because of their risk for recurrent falls (Jehu et al., 2021b) and distinct needs, such as forgetfulness (Jehu et al., 2024b), planning difficulties (Hauer et al., 2006), and apathy (Clarke et al., 2008). PLWD also commonly experience social isolation (Shen et al., 2022), poorer physical function (Jehu et al., 2024a), worsened behavioral and psychological symptoms (Cerejeira et al., 2012), poorer quality of life (Cerejeira et al., 2012), and reduced access to healthcare (Mitchell et al., 2016) compared to older adults without dementia. Designing efficacious interventions to improve health outcomes among PLWD is a frontline priority (Corriveau et al., 2017).

## Potential benefits of dance interventions for PLWD

Dance can be particularly beneficial as a lifestyle intervention for PLWD through promoting social networking and enjoyment while boosting physical activity (Kshtriya et al., 2015). The aesthetic and creative aspects of dance may promote greater physiological arousal than aerobic exercise at a similar intensity (Fontanesi and DeSouza, 2020), but this should be confirmed by further studies with larger samples. Music and dance also encourage spontaneous rhythmic coupling between sensory and motor systems (Krotinger and Loui, 2021), and facilitate self-expression and the rediscovery of skills such as moving and singing as means of communicating (Hamill et al., 2012; Kshtriya et al., 2015).

Dementia is a progressive neurological disease that affects activities of daily living, while mild cognitive impairment is defined as cognitive decline greater than that expected for a person's age and education level, without significant functional impairment (Gauthier et al., 2006). Although dance has potential as a therapeutic intervention for PLWD, research on this population is scarce. A systematic review of dance interventions for PLWD (Karkou et al., 2023) found only one randomized controlled trial, which also included those with mild cognitive impairment (Ho et al., 2020), possibly masking between-group differences in responsiveness. This study found that dance improved loneliness, mood, physical functioning, and stress relative to a wait-list control group (Ho et al., 2020). Another randomized controlled trial comparing 2 months of Latin dance with a wait-list control found no differences in physical activity, fitness, or sedentary behavior, possibly due to the small sample size of *n* = 21 PLWD (Aguiñaga and Marquez, 2019). Other non-randomized controlled trials have indicated that dance is feasible in residential care facilities, but found no changes post-intervention, possibly due to small sample sizes (*n* < 30) (Hokkanen et al., 2008, 2003). One case study showed improved physical function following a 12-week salsa dance intervention in an individual with Alzheimer's disease (Abreu and Hartley, 2013). A quasi-experimental study comparing a 10-week dual-task intervention with an Iranian dance intervention found improvements in gait parameters among n=38 female PLWD (Ghadiri et al., 2022). Qualitative and non-randomized research has observed positive emotional reactions during dance among PLWD (Palo-Bengtsson and Ekman, 2002; Hamill et al., 2012; Guzmán-García et al., 2013; Bumanis and Yoder, 1988). Other research has indicated that PLWD exhibit procedural learning of the dance movements throughout the intervention (Rösler et al., 2002). Despite promising findings, there is a need to extend beyond feasibility studies and investigate dance interventions with adequate power and an active comparison group. General recommendations for exercise dosing exist for PLWD (Bushman, 2021), but further work should establish precise evidence-based exercise guidelines to improve outcomes in PLWD. No evidence-based dance guidelines exist to improve outcomes in PLWD. This opinion paper focuses on tailoring dance interventions for PLWD to increase uptake and improve treatment design.

## Key considerations for dance for PLWD

### Accessibility of dance

Researchers and dance instructors should make dance accessible, emphasizing participation over performance, to foster creativity and awareness of the body rather than accuracy (Hamill et al., 2012). PLWD may find dance easier in a partnered than group format because the former involves person-to-person interaction and connection (Abraham et al., 2024). Partnered dance involves leader and follower roles. The “leader” role, a proxy for internally generated movements, conveys direction, timing, and amplitude of steps with tactile cues, while the “follower” role, a proxy for externally generated movements, detects and responds to the leader's tactile cues (Abraham et al., 2024). The leader role is more cognitively demanding, requiring participants to remember specific movement sequences and choose appropriate next steps, while the follower role receives and responds to ongoing non-verbal cues. It may therefore be more beneficial to have PLWD in the less cognitively demanding follower role. Instructors should provide opportunities to engage in person-to-person contact and move together as a unit; such opportunities promote re-attachment and connection by overcoming communication difficulties, thereby building a socially cohesive environment (Hamill et al., 2012). Depending on dementia symptoms, such as agitation, PLWD may prefer non-partnered dance. Regardless of the dance type, participants should be encouraged to express themselves freely and spontaneously (Hamill et al., 2012). Instructors should wear name tags and simulate social interactions with group members while dancing (Hamill et al., 2012). We recommend regular reassurance, especially in times of confusion (Izquierdo et al., 2021). To foster meaningful progress in PLWD, we also recommend short and simple exercise instructions and mirroring techniques (i.e., performing dance moves with the PLWD enabling them to copy movements) (Izquierdo et al., 2021).

### Individual adaptations

Adaptations to changing functional capacity levels should be considered throughout the intervention, to account for physical and functional limitations and secondary medical complications (Cipriani et al., 2020). Dementia is a non-linear progressive neurological disease that varies across individuals (Sachdev et al., 2014); thus, simpler instructions may be needed for PLWD as cognitive decline progresses. Cognitive, physical, and functional impairments may impact engagement and adherence levels; consequently, instructors should tailor dance programs to individuals' abilities. Conversely, PLWD may experience cognitive, physical, and functional improvements during interventions (Bracco et al., 2023); thus, complexity should be adjusted such that dance programs remain adequately challenging. Ongoing consultation with the PLWD's healthcare team (e.g., neuropsychologist), who are aware of the level of functional impairment, is important for appropriate dance prescription throughout the intervention (ACSM, 2021).

### Caregiver support

Dance interventions seem to decrease caregiver burden (Wharton et al., 2021), and caregiver support is needed for PLWD to successfully engage in interventions. Reminders to attend classes can benefit PLWD who may lack an advocate and forget to attend (Portacolone et al., 2023), or may be aware of their own cognitive decline and avoid social activities due to embarrassment and stigmatization (Ho et al., 2021; Zhu et al., 2023). Partnered dance may be more beneficial for PLWD because a caregiver or instructor could act as a partner to cue for dance moves, help with spotting to reduce fall risk, and increase supervision for those who wander. However, partnered dance would likely increase intervention costs relative to group dance as each PLWD would need a cognitively intact partner (e.g., instructor/assistant).

### Stakeholder input

Interventions should be designed with key stakeholders (e.g., caregivers, residential care facility staff, patients, and community advisory board) to ensure adequate training, resources, and support for PLWD in the community and residential care (Jehu et al., 2023b). Residential care facility staff have been hesitant to implement physical activity programs among PLWD due to therapeutic pessimism (Knaak et al., 2017), perceived risk, low belief in their utility, insufficient training and support, workload concerns, and high staff turnover (Wylie et al., 2022). Among PLWD, barriers to physical activity may be attitudinal or physical and include disliking physical activity, lacking experience of being physically active, pre-existing chronic conditions, sickness, holidays, and caregiver factors such as unavailability and health concerns (Suttanon et al., 2012). While residential care facility routines and negative attitudes about physical activity have reduced physical activity promotion in many facilities (Wylie et al., 2022), behavior change is possible among facility staff, caregivers, and PLWD (Low et al., 2015). For community-dwelling PLWD, it is important to support caregivers in delivering dance interventions to reduce burnout, such as providing educational resources, training, and regular phone calls (Jehu et al., 2023a). Several organizational factors should be considered when designing interventions, including ensuring that support (e.g., escorting PLWD to exercise, scheduling) and resources (e.g., staff) are available from the organization, as well as training researchers and dance instructors on the importance of dance interventions for PLWD, dance and dementia communication techniques, and safety (e.g., training for Cardiopulmonary Resuscitation and spotting) (Demers et al., 2015). Including PLWD and a community advisory board as members of the team can enable facilitators, avoid barriers, and promote generalizability to the larger population.

## Considerations for choice of dance style and interventional targets

Selecting appropriate outcomes depending on individual needs, the type of dance, and dementia subtypes should be considered in the intervention design (Jehu et al., 2023a). For example, if the therapeutic target is to improve cardiovascular health, the current guidelines for prescribing exercise intensity should be followed (Riebe et al., 2015), and heart rate should be monitored throughout the intervention. Additional research is needed to identify optimal exercise intensities for improving health outcomes in specific populations, such as those with vascular dementia (Barnes and Corkery, 2018). If the therapeutic target is improving cognitive health, dance types involving visual-spatial cognition, memory, and planning, such as Argentinian Tango, may be appropriate, especially for people with Alzheimer's disease (Wharton et al., 2021). The dance style should be carefully selected for the target population, as even low-intensity dance could be challenging for deconditioned PLWD. The complexity of dance moves should be considered depending on dementia severity, as dance movements are generally more complicated than other types of exercise. To date, no studies have compared the effects of different doses of dance among PLWD for any outcome (Rice et al., 2024). Such research would provide insight into the frequency, intensity, and duration of dance required to achieve optimal benefits for PLWD.

It is important to standardize person-centered valid and reliable outcome measures that are sensitive to change in dementia to increase comparability across studies. In a recent scoping review of more than 40 different types of dance intervention, over 50 cognitive measures and 30 mobility measures were identified, highlighting the lack of consistency in the standardization of outcomes (Rice et al., 2024). The outcome measures should also be feasible for PLWD. For example, previous work has documented substantial missing data due to poor adherence to wrist-worn activity monitors (Jehu et al., 2024b). Minor protocol changes (e.g., placing activity monitors on the lower back rather than the wrist) may increase adherence. A recent systematic review and consensus statement recommends a specific set of outcome measures for PLWD across cognition, activities of daily living, biological markers, neuropsychiatric symptoms, quality of life, and global domains (Webster et al., 2017). Dance researchers may want to provide similar recommendations in a consensus statement and add important measures such as balance, gait, falls, and social determinants of health (Jehu and Skelton, 2024, 2023; Jehu et al., 2021a). Researchers may also consider additional outcome measures depending on their research question, such as caregiver burden. Table 1 outlines a summary of our recommendations.

**Table 1:** Summary of recommendations for dance intervention design and measurement outcomes.

**General recommendations for structuring interventions for dementia**	
✓ Simple instructions ✓ Slow music tempo ✓ Mirroring technique ✓ Repetitive choreography ✓ Partnered dance for spotting ✓ Familiar music to enhance mood and enjoyment, and support reminiscence ✓ Reminders to attend dance class (e.g., on their door on the exercise day) ✓ Appropriate trainer-to-participant ratio	✓ Name tags on instructors and participants ✓ Schedule dance interventions in the morning to avoid sundowning ✓ Schedule around meals and other events at the residential care facilities to increase adherence ✓ Have a champion to support adherence ✓ Design the intervention based on feedback from key stakeholders ✓ Communicate with PLWD and their caregivers with empathy, listen to their concerns, and validate their feelings
**Considerations for choosing the type of dance intervention in dementia**	
Select the dance style depending on the therapeutic target ✓ Select feasible, valid, reliable, and responsive outcome measures in PLWD	More research is needed to determine the optimal dose of dance in PLWD for all outcomes

## Future research directions

Preliminary research indicates potential cognitive, motor, and psychological benefits of dance interventions for PLWD (Balbim et al., 2022; Cezar et al., 2021). More rigorous research with larger sample sizes is needed. Involving PLWD, their caregivers, and clinicians in designing tailored and accessible dance interventions, as well as selecting appropriate outcomes, may increase relevance for this population. Dance intervention design should be tailored to neuropsychological and behavioral symptoms associated with specific dementia subtypes, such as addressing wandering for those with Alzheimer's disease or providing external cues for those with Parkinson's-related dementias. Our recommendations are more specifically for PLWD who are still able to follow instructions; further research is needed to involve those with more severe dementia. A better understanding of the impact of dance interventions on cognitive, motor, and psychological outcomes may inform targeted treatment and monitoring strategies in PLWD.
